# Large-Scale Identification and Characterization Analysis of *VQ* Family Genes in Plants, Especially Gymnosperms

**DOI:** 10.3390/ijms241914968

**Published:** 2023-10-06

**Authors:** Jinfu Tian, Jiahui Zhang, Frédéric Francis

**Affiliations:** 1 Functional and Evolutionary Entomology, Gembloux Agro-Bio Tech, University of Liège, 5030 Gembloux, Belgium; jinfu.tian@student.uliege.be (J.T.);; 2 Institute of Crop Sciences, Chinese Academy of Agricultural Sciences (CAAS), Beijing 100081, China

**Keywords:** large-scale identification, clear graphical presentation, *VQ* family genes, molecular and evolutionary features, gymnosperms

## Abstract

VQ motif-containing (VQ) proteins are a class of transcription regulatory cofactors widely present in plants, playing crucial roles in growth and development, stress response, and defense. Although there have been some reports on the member identification and functional research of *VQ* genes in some plants, there is still a lack of large-scale identification and clear graphical presentation of their basic characterization information to help us to better understand this family. Especially in gymnosperms, the *VQ* family genes and their evolutionary relationships have not yet been reported. In this study, we systematically identified 2469 *VQ* genes from 56 plant species, including bryophytes, gymnosperms, and angiosperms, and analyzed their molecular and evolutionary features. We found that amino acids are only highly conserved in the VQ domain, while other positions are relatively variable; most *VQ* genes encode relatively small proteins and do not have introns. The GC content in Poaceae plants is the highest (up to 70%); these VQ proteins can be divided into nine subgroups. In particular, we analyzed the molecular characteristics, chromosome distribution, duplication events, and expression levels of *VQ* genes in three gymnosperms: *Ginkgo biloba*, *Taxus chinensis*, and *Pinus tabuliformis*. In gymnosperms, *VQ* genes are classified into 11 groups, with highly similar motifs in each group; most VQ proteins have less than 300 amino acids and are predicted to be located in nucleus. Tandem duplication is an important driving force for the expansion of the *VQ* gene family, and the evolutionary processes of most *VQ* genes and duplication events are relatively independent; some candidate *VQ* genes are preliminarily screened, and they are likely to be involved in plant growth and stress and defense responses. These results provide detailed information and powerful references for further understanding and utilizing the *VQ* family genes in various plants.

## 1. Introduction

VQ motif-containing (VQ) protein is known as a transcription regulatory cofactor for interacting with transcription factors (TFs) to regulate gene expression. In 2002, *AtVQ23*/*AtSIB1* was first identified in *Arabidopsis* [1]. Subsequently, the *VQ* genes were successively identified via bioinformatics and experimental methods in various plants, such as rice, soybean, maize, grape, and wheat [2,3].

VQ proteins contain the highly conserved VQ motif (FxxxVQxhTG: x represents arbitrary amino acid, and h represents hydrophobic amino acid) [2,4]. The three terminal amino acids in the VQ motif may have different types in different plants. For example, six types (LTG, FTG, VTG, YTG, LTS, and LTD) were identified in *Arabidopsis*, four types (LTG, FTG, VTG, and ITG) were identified in rice, and five types (LTG, FTG, VTG, ITG, and VMA) were identified in wheat [3]. Most *VQ* genes in higher plants have no introns and encode relatively small proteins with less than 300 amino acids [2,4]. The lack of introns in these gene sequences makes transcription and translation more efficient, resulting in the production of these small proteins. Most VQ proteins are located in the nucleus, with a few found in the chloroplast and cytoplasm [5,6,7]. Moreover, it has been reported that VQ motif is related to the interaction with WRKY [2]. These basic characteristics provide a certain reference for us to understand and identify *VQ* family genes.

Jasmonic acid (JA), salicylic acid (SA), and abscisic acid (ABA) are important hormone signaling molecules in plants, which are widely involved in plant growth and development, disease resistance, and stress responses [8,9,10,11]. Research has shown that the expression of many *VQ* genes in different plants is induced or inhibited by JA, SA, or ABA hormones, as well as treatments such as pathogens, drought, and salt [3,5,6]. This finding indicates that the *VQ* gene plays an important role in complex signaling pathways in response to JA, SA, and ABA hormones and various stresses. In addition, WRKY, MAPK, and calmodulin (CaM) have been reported to play very important roles in plant life processes and responses to external stimuli [12,13,14,15,16]. By interacting with proteins such as CaM, WRKY, and MAPK, VQ proteins can mediate plant growth and development, as well as defense responses to biotic and abiotic stresses [2,5,17,18,19,20].

Studies have shown that VQ proteins are involved in growth and development. For example, in *Arabidopsis*, the loss-of-function mutants of *AtVQ8* showed yellow–green leaves and delayed growth, while plants overexpressing *AtVQ17*, *AtVQ18*, or *AtVQ22* showed a stunted phenotype with severely inhibited growth [5]. AtVQ14/IKU1 interacts with WRKY10/MINI3 to regulate the development of seed [21,22]. AtVQ20 interacts with WRKY2 and WRKY34 to regulate the expression of downstream *MYB* genes, thereby affecting male fertility [23]. AtVQ18 or AtVQ26 can interact with ABI5 and inhibit its transcriptional activation ability to negatively regulate ABA responses during seed germination and seedling establishment [24]. AtVQ29 interacts with PIF1 to enhance its transcriptional activation activity, regulating the elongation of hypocotyls under different spectra [25].

VQ proteins are involved in the responses to abiotic stresses. For example, AtVQ9 interacts with WRKY8 to regulate the expression of *AtRD29A*, negatively regulating plant salt tolerance [26]. AtVQ15 interacts with CaM, WRKY25, and WRKY51 to regulate plant tolerance to osmotic stress [5,17]. Bamboo PeVQ28 interacts with PeWRKY83 to promote the expression of ABA-related genes and positively regulates salt tolerance in plants [27]. Ectopic overexpression of tomato *SlVQ6* in *Arabidopsis* showed decreased high-temperature tolerance [28]. The hypermorphic mutant of *AtVQ10* exhibited enhanced meristem development, increased tolerance to oxidative stress, and reduced sensitivity to NO [29].

VQ proteins are involved in the response to biotic stresses. AtVQ4/MVQ1 is phosphorylated by MPK3/6, which, in turn, affects the interaction between VQ and WRKY to finely regulate the immune response in *Arabidopsis* [19]. Both AtVQ16/SIB2 and AtVQ23/SIB1 can bind to WRKY33 to regulate plant defense against *Botrytis cinerea* [5,30]. AtVQ21/MKS1 interacts with MPK4 and WRKY25/33 to regulate the expression of downstream genes. The overexpression of *AtVQ21*/*MKS1* significantly increases resistance to *Pseudomonas syringae* by participating in the SA pathway [31], while reducing resistance to *B. cinerea* through the negative regulation of the JA pathway [32,33]. AtVQ22/JAV1 interacts with Ca^2+^/CaM, JUL1, JAZ8, and WRKY51 to jointly regulate JA synthesis, leading to rapid JA burst and activating plant defense [18,34]. OsVQ25 interacts with OsPUB73 and OsWRKY53 to balance the broad-spectrum disease resistance and growth of rice [35].

Some gymnosperms have high ornamental, medicinal, and economic value. They are also important materials for studying plant evolution. However, the research into the *VQ* family genes in gymnosperms has not yet been reported. Many gymnosperms have large genomes and numerous repetitive sequences, making it difficult to assemble a complete genome. But, in recent years, with the advancement in technology, some gymnosperms have gradually completed genome sequencing and assembly [36,37,38,39,40]. In view of the prominent role of *VQ* genes in the growth and development and the responses to environmental stress in angiosperms such as *Arabidopsis* and rice, *VQ* genes may also participate in various life processes in gymnosperms. Therefore, studying the morphology and evolution of *VQ* genes in gymnosperms will be very interesting and meaningful.

Although *VQ* genes have been structurally and functionally identified in some plants, there is a lack of systematic analysis and intuitive display to clearly show their characteristics in a wider range of plants. Here, in order to identify the detailed information on *VQ* family genes in the plant kingdom, we characterized a total of 2469 *VQ* genes from 56 plant species, including bryophytes, gymnosperms, and angiosperms. We carried out a comprehensive bioinformatics analysis, including conserved motifs, basic molecular characterization, and systemic clustering. Importantly, for gymnosperms, we selected *Ginkgo biloba*, *Taxus chinensis*, and *Pinus tabuliformis* as the research objects; identified their *VQ* gene members; and analyzed their molecular features, gene structures, subcellular locations, chromosome distributions, duplication events, expression levels, synteny blocks, and evolutionary comparisons. Our results provide fundamental information about the characterization and evolution of *VQ* genes in gymnosperms and angiosperms, which will be valuable for further research into their biological functions and working mechanisms.

## 2. Results

### 2.1. Members Identification and Conserved Motif Analysis of VQ Genes in Multiple Plants

**Figure 1:**
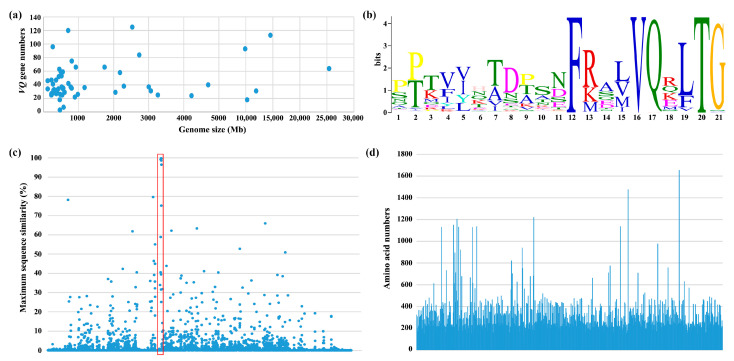
Members identification and sequence characteristics of *VQ* genes. (**a**) The relationship between genome size and *VQ* gene numbers in 56 plant species. (**b**) The conserved VQ motif. (**c**) The maximum sequence similarity in each alignment site. The red frame is the region of the VQ motif, and the X-axis represents alignment site. (**d**) The length of VQ proteins. The X-axis represents 2469 VQ proteins, and the Y-axis shows amino acid numbers of VQ protein.

The Hidden Markov Model (HMM) of the VQ motif (PF05678) was used to search the coding proteins for putative *VQ* genes in each species. After manually removing invalid entries and validating the search results, we systematically identified a total of 2469 *VQ* family genes from 56 plant species, including 3 bryophytes, 3 gymnosperms, and 50 angiosperms. Moreover, their basic information, such as VQ protein sequences (Table S1), chromosome ploidy, genome size, and numbers of total coding genes or *VQ* genes (Table S2), were summarized and listed for each species. We found that there is not any necessary relationship between genome size and *VQ* gene numbers (Figure 1a), consistent with previous studies [4,41].

We scanned 2469 VQ proteins and found the most conserved motif is FxxxVQxhTG, where three main terminal amino acids, namely LTG, FTG, and VTG (Figure 1b), are located. The core element ‘VQ’ in FxxxVQxhTG, is slightly changed in some plants, such as FxxxVHxhTG (Table S1), which is in agreement with a previous study [3]. Studies have shown that many VQ proteins contain single- or dual-component nuclear localization signals, and some also contain chloroplast targeting signals [5,6,7]. Moreover, some VQ proteins were reported as containing calmodulin (CaM) binding domains, such as AtVQ15 and AtVQ22 [17,18], or MAPK phosphorylation sites [19]. We predicted conservative motifs using MEME online tool. The top 20 conserved motifs were listed in Figure S1, and these motifs may be related to protein localization and protein interactions. We searched the database for these motifs using the Tomtom program within MEME online tool and did not find any clear functional annotations. Given that most VQ proteins were predicted to be localized in the nucleus, we speculated that there should be motifs associated with nuclear localization. It is known that proteins such as JAV1 and OsVQ25 are located in the nucleus [18,35]. Therefore, we used NLStradamus and PSORT online tools to predict their nuclear localization signals. We found that Motif 4 is directly related to the nuclear localization of these proteins (Figures S1 and S2). Interestingly, we also found a significant overlap between the CaM binding domain [17,18] and Motif 4, suggesting that Motif 4 may both guide nuclear localization and participate in interactions with CaM in these proteins.

### 2.2. Sequence Similarities, Length, Introns, and GC Content Analysis of VQ Genes in Multiple Plants

After performing multiple sequence alignment using MAFFT (v7.511) software on 2469 VQ proteins, all VQ proteins had an equal alignment length, and the proportion of identical amino acids at each aligned site was calculated. The highest ratio is the maximum sequence similarity of this matching point. This observation can help us to clearly see the overall sequence similarity of *VQ* family genes in plants. We found that the proportion of alignment sites with maximum sequence similarity ≤ 20% is 99.2%, and except for the VQ motif, the amino acid sequences at other positions are very variable (Figure 1c). Overall, the similarity of VQ proteins is low, but there are also some amino acid sites showing slightly higher similarity. Therefore, for the certain *VQ* gene, it is still possible to find relatively homologous genes in different species according to their sequence similarity.

**Figure 2:**
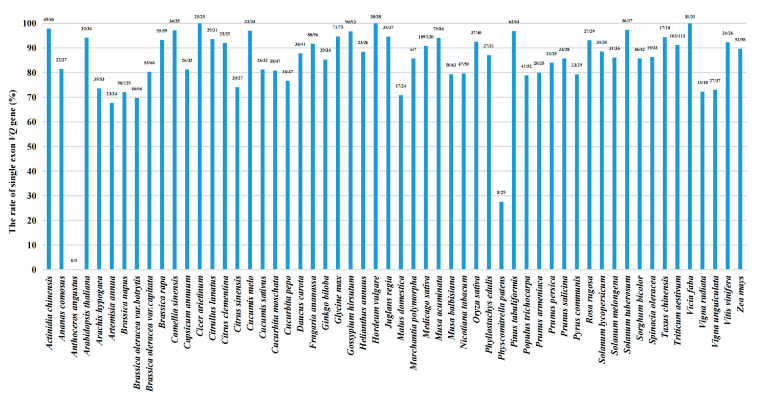
The ratio of single exon (intron-free) *VQ* genes. The labels above the column mean ‘numbers of single-exon *VQ* genes/numbers of all *VQ* genes’.

We conducted the basic statistics regarding these 2469 *VQ* genes. Most *VQ* genes encode relatively small proteins, with 83% and 93% of them having less than 300 and 400 amino acids, respectively (Figure 1d), almost consistent with previous studies [2,4]. In species with multiple *VQ* genes, moss (*Physcomitrella patens*) keeps high proportion (more than 70%) of intron-containing *VQ* genes, while most *VQ* genes in higher plants do not have introns, no matter their angiosperms or gymnosperms (Figure 2). It has previously been reported that most *VQ* genes in moss (*Physcomitrella patens*) have introns [28], but interestingly, we inadvertently found that in different tea varieties (*Camellia sinensis*), such as ‘Tieguanyin’ (used in this study), ‘Longjing 43’, ’Shuchazao’, ‘Yunkang 10’, and ‘Biyun’, the ratios of intron-free *VQ* genes are mostly high, except for ‘Longjing 43’, whose proportion is actually as low as 15% (Table S3).

**Figure 3:**
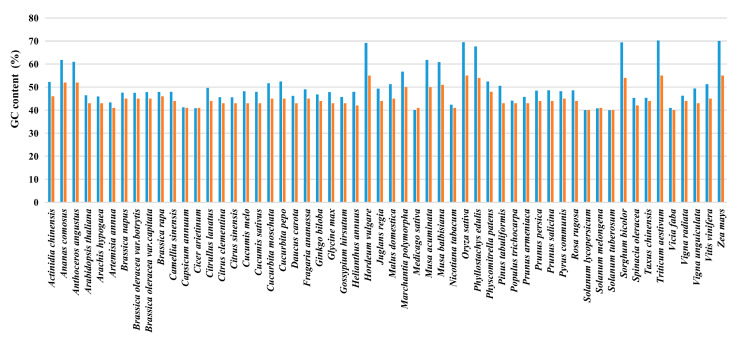
The average GC content of genes in the coding sequence. The blue column is just for *VQ* genes, and the red column is for all coding genes in each species.

Furthermore, we calculated GC content in the coding region of the *VQ* gene. Interestingly, we found that the average GC content in the coding sequence of *VQ* genes among all species is greater than 40%; in commelinids, including Poales and Musa plants, GC content is more than 60%. In Poaceae plants, such as bamboo, sorghum, rice, maize, barley, and wheat, the GC content is up to 70%, which is much higher than the average level of the species (55%), making it a relatively special occurrence in plants (Figure 3). This finding suggests that *VQ* genes are more stable in Poaceae plants and may play more prominent roles in evolution and function.

### 2.3. Molecular Features and Gene Structure Analysis of VQ Genes in Gymnosperms

**Figure 4:**
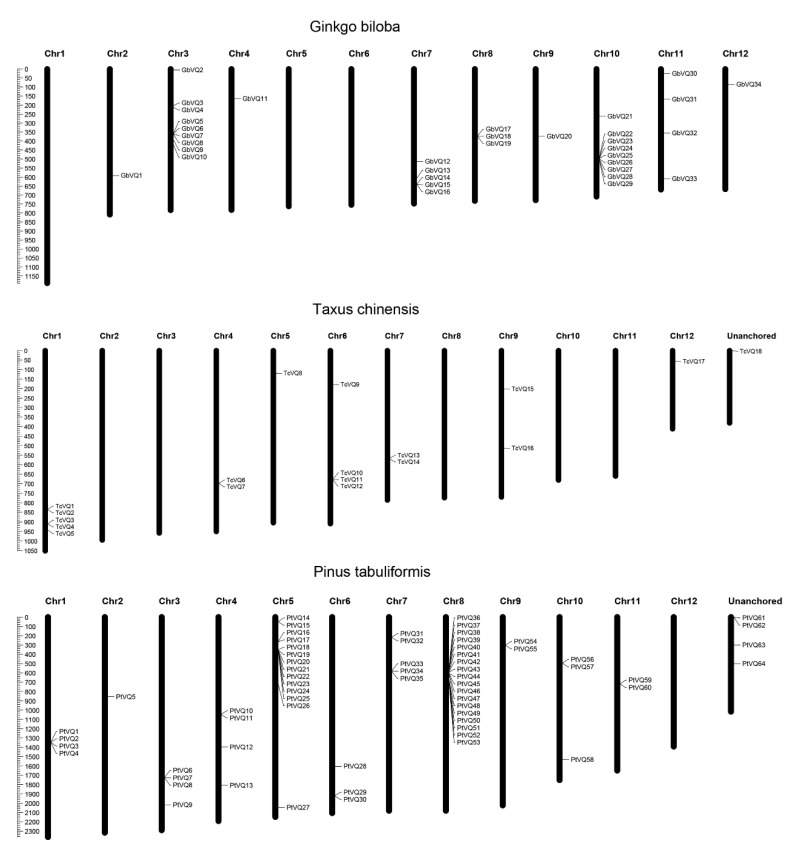
Chromosomal distribution of *VQ* genes in three gymnosperms. Chromosome numbers are listed at the top. The length of the chromosome is displayed in megabase (Mb) scale.

In total, 34, 18, and 64 *VQ* genes were identified in *Ginkgo biloba*, *Taxus chinensis*, and *Pinus tabuliformis*, respectively, designated as *GbVQ1* to *GbVQ34*, *TcVQ1* to *TcVQ18*, and *PtVQ1* to *PtVQ64* based on their chromosomal physical location (Table 1 and Table S4). In order to clearly see the distribution of *VQ* genes on chromosomes, MapChart (v2.3) software was used to produce the chromosome map on chromosomes according to the detailed location information of *VQ* genes in the GFF file. These *VQ* genes are not evenly distributed across different chromosomes, and on certain chromosomes, there is no *VQ* gene distribution (Figure 4). Among them, chromosome 3 and chromosome 10 have the largest number of *VQ* genes in *Ginkgo biloba* (*n* = 9, *n* = 9)*,* chromosome 1 has the largest number of *VQ* genes in *Taxus chinensis* (*n* = 5), and chromosome 5 and chromosome 8 have the largest number of *VQ* genes in *Pinus tabuliformis* (*n* = 14, *n* = 18). In addition, we noticed that there are multiple *VQ* genes stacking within specific chromosomal regions, which may be due to the huge repetitive sequence or genome replication events of gymnosperms.

In gymnosperms, we analyzed the basic molecular features of *VQ* genes, and the detailed data for each *VQ* gene, including gene ID, chromosome position, protein length, motif type, isoelectric point, molecular weight, and subcellular localization, were shown in Table S4. The length of their encoded VQ protein ranges from 126 amino acids (GbVQ19) to 519 amino acids (GbVQ32), with an average of 283 amino acids, in *Ginkgo biloba*; ranges from 110 amino acids (TcVQ12) to 243 amino acids (TcVQ9), with an average of 165 amino acids, in *Taxus chinensis*; and ranges from 121 amino acids (PtVQ11) to 709 amino acids (PtVQ13), with an average 260 amino acids, in *Pinus tabuliformis*. The analysis of physiochemical properties further revealed that VQ proteins are widely varied in molecular weight (MW), ranging from 14.43 (GbVQ19) to 54.51 kDa (GbVQ32), with an average of 30.86 kDa, in *Ginkgo biloba*,; ranging from 12.26 (TcVQ12) to 54.51 kDa (TcVQ9), with an average of 18.37 kDa, in *Taxus chinensis*; and ranging from 13.08 (PtVQ11) to 76.10 kDa (PtVQ13), with an average of 28.09 kDa, in *Pinus tabuliformis*. The isoelectric point (pI) of these VQ proteins varies between 4.83 (GbVQ21) and 10.43 (GbVQ3) in *Ginkgo biloba* (average 7.67), 5.06 (TcVQ5) and 10.27 (TcVQ15) in *Taxus chinensis* (average 8.55), and 5.07 (PtVQ24) and 11.03 (PtVQ31) in *Pinus tabuliformis* (average 8.50) (Table 1 and Table S4).

In addition, we identified four VQ motif types (LTG, FTG, VTG, and YTG) in *Ginkgo biloba*, four VQ motif types (LTG, FTG, VTG, and FTA) in *Taxus chinensis*, and four VQ motif types (LTG, FTG, MTG, and VTG) in *Pinus tabuliformis.* Most VQ proteins belong to the LTG type (23/34), with eight FTG type (8/34), two VTG type (2/34), and one YTG type (1/34) found in *Ginkgo biloba*; many LTG type (13/18), three FTG type (3/18), one VTG type (1/18), and one FTA type (1/18) found in *Taxus chinensis*; and many LTG type (36/64), twenty-two FTG type (22/64), four MTG type (4/64), and two VTG type (2/64) found in *Pinus tabuliformis* (Table 1 and Table S4). We also observed slight changes in the core VQ motif from FxxxVQxhTG to FxxxVExhTG (PtVQ37, PtVQ38, PtVQ39) and FxxxVHxhTG (PtVQ28) in *Pinus tabuliformis* (Table S1). The subcellular localization analysis revealed that most VQ proteins were predicted to be located in the nucleus, while a few were predicted to be located in the cytoplasm/nucleus (Table 1 and Table S4).

**Figure 5:**
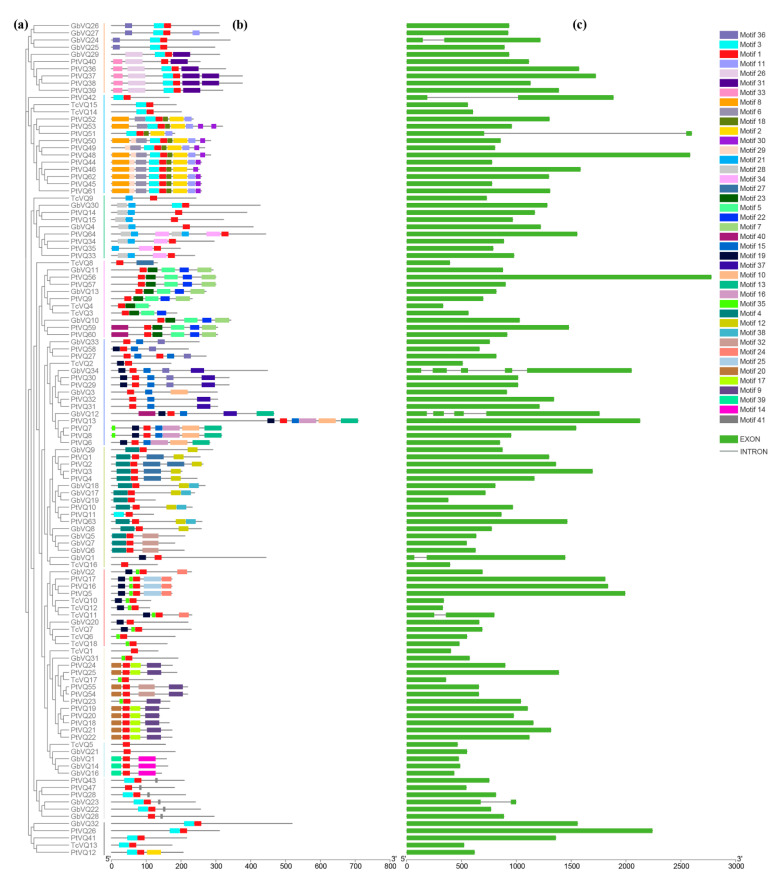
Phylogenetic tree, conserved motifs, and gene structure of VQ protein in three gymnosperms. (**a**) Phylogenetic tree of 116 VQ proteins in three gymnosperms based on the results of sequence alignment. (**b**) A total of 41 conserved motifs of the VQ protein. Each specific motif is indicated by a different colored box. (**c**) Gene structure (exon–intron) of the *VQ* genes. Exons are indicated by green rectangles, and lines connecting two exons represent introns.

Gene structure can provide more information about the evolutionary relationship within a gene family. We conducted a systematic clustering and gene structure analysis of the *VQ* genes in *Ginkgo biloba*, *Taxus chinensis*, and *Pinus tabuliformis*. We detected 41 conservative motifs using MEME online tool, and we can clearly see the differences between these *VQ* genes. Motif 1 was identified as the core motif that comprises the VQ domain, which was included in all VQ proteins. It is noteworthy that *VQ* genes with closer clustering relationships have almost similar conserved motifs, indicating that the phylogenetic classification is relatively reliable and proteins in the same group maybe perform similar functions (Figure 5a,b). We created an exon/intron structure map based on the location information of each *VQ* gene in the GFF file. The structure of *VQ* genes was analyzed, and we found 85%, 94%, and 96% of *VQ* genes to have no introns in *Ginkgo biloba*, *Taxus chinensis*, and *Pinus tabuliformis*, respectively (Figure 5c). These findings indicate that most *VQ* genes do not contain introns in gymnosperms, which is similar to the results of angiosperms [2].

### 2.4. Gene Duplication and Collinearity Analysis of VQ Genes in Gymnosperms

Gene duplication is an important event contributing to genome evolution, and it is also an important factor in the expansion of the gene family. It is primarily divided into tandem duplication, segmental duplication, dispersed duplication, and proximal duplication [42]. To better understand the evolutionary mechanisms of *VQ* genes in gymnosperms, the duplication events of the *VQ* family gene were evaluated. Following the BLAST and MCScanX results, among the 34 *GbVQs*, a total of 10 members and 8 gene pairs participated in duplication events in *Ginkgo biloba*; among the 18 *TcVQs*, a total of 3 members and 2 gene pairs participated in duplication events in *Taxus chinensis*, and among the 64 *PtVQs*, a total of 18 members and 33 gene pairs participated in duplication events in *Pinus tabuliformis* (Table 2). *Ginkgo biloba* exhibited five tandem duplication events (5/8), two proximal duplication events (2/8), and one segmental duplication events (1/8); *Taxus chinensis* exhibited two proximal duplication events (2/2); and *Pinus tabuliformis* exhibited fourteen dispersed duplication events (14/33), ten tandem duplication events (10/33), and nine proximal duplication events (9/33). In these three gymnosperms, there is an interesting phenomenon: although the distance between two or more *VQ* gene pairs is a little bigger than 200 kb (here marked as proximal duplication), they are continuously arranged coding genes in one genome block; therefore, this type of gene pair may also be named as tandem duplication (Figure 4 and Table 2). These results suggest that tandem duplication is important to expand the *VQ* family gene in gymnosperms. While whole-genome duplication (WGD) played a critical role in adaptive evolution in angiosperms [43], few recent WGD events were found in extant gymnosperms [36,37,38,39,40,44], which indicates that these duplicate *VQ* gene pairs were evolved from independent duplication events or derived from older ancestors.

The selective evolutionary pressure on all *VQ* gene pairs was investigated by calculating the Ka, Ks, and Ka/Ks ratios of the duplication events. The Ka/Ks values of most duplicated gene pairs (5/8) are less than 1.0, those of two gene pairs (2/8) are slightly greater than 1.0, and that of one gene pair (3/8) (segmental duplication events) is unable to calculate a valid value using KaKs_Calculator 2.0 software in *Ginkgo biloba*. One duplicated gene pair (1/2) is slightly greater 1.0, and one gene pair (1/2) is unable to calculate a valid value in *Taxus chinensis*; most duplicated gene pairs (21/33) are less than 1.0, four gene pairs (4/33) are more than 1.0, one gene pair (1/33) is unable to calculate a valid value, and seven duplicated gene pairs (7/33) have Ka = Ks = 0, which means these two genes have no difference in their coding region in *Pinus tabuliformis* (Table 2). The Ka/Ks values of the majority of *VQ* gene pairs in gymnosperms are less than 1.0, indicating they have mainly undergone purifying selection pressures during evolution process. The Ka/Ks values of some duplicated gene pairs are greater than 1.0, which shows the presence of positive selection pressure.

**Figure 6:**
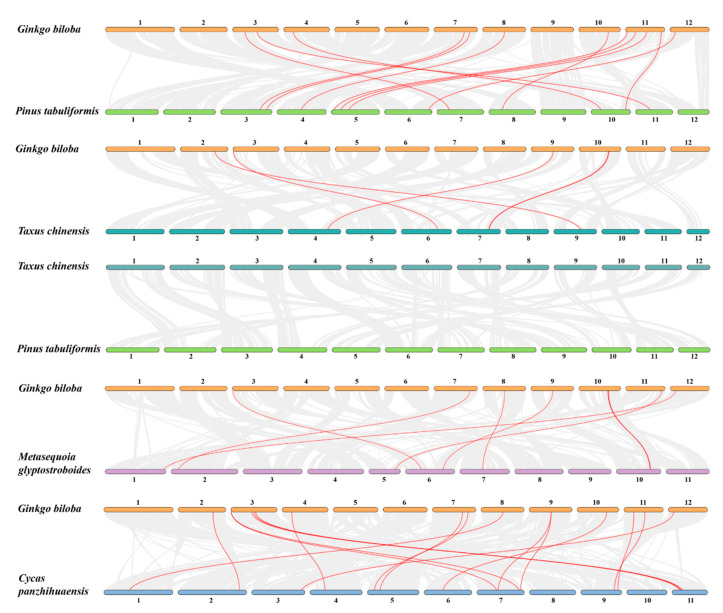
Collinear analysis between different gymnosperms. The grey lines in the background represent the collinear regions between different plant genomes, while the red lines highlight the collinear *VQ* gene pairs. The numbers represent chromosomes.

To gain insight into the evolution of *VQ* genes in gymnosperms, the collinear blocks were searched in the chromosomes using MCScanX software. *Ginkgo biloba* and *Pinus tabulaeformis* have the most collinear blocks at the whole genome level, followed by *Ginkgo biloba* and *Taxus chinensis* and *Taxus chinensis* and *Pinus tabuliformis*. This finding indicates that compared to *Taxus chinensis*, *Ginkgo biloba* has a higher similarity and closer evolutionary relationship with *Pinus tabuliformis.* Similarly, the *VQ* gene pairs in these collinear blocks are the most between *Ginkgo biloba* and *Pinus tabuliformis* (*n* = 12), followed by *Ginkgo biloba* and *Taxus chinensis* (*n* = 5); however, *Taxus chinensis* and *Pinus tabuliformis* do not have orthologous *VQ* gene pairs (*n* = 0) in fewer collinear blocks (Figure 6). Among all *VQ* gene pairs mentioned above (*n* = 17), only one *VQ* gene (*GbVQ29*, *TcVQ13*, and *PtVQ38*) was shared by these three gymnosperms. In order to better see the conservatism of these *VQ* genes in different gymnosperms, we introduced the collinearity comparison with another two gymnosperms: *Cycas panzhihuaensis* [38] and *Metasequoia glyptostroboides* [40]. We found that this *VQ* gene and its related collinear block also existed in *Metasequoia glyptostroboides* and *Cycas panzhihuaensis*. Moreover, in all collinear blocks, we found five other *VQ* genes (*GbVQ2*, *GbVQ13*, *GbVQ17*, *GbVQ20*, and *GbVQ34*), most of which contain collinear *VQ* genes in these five gymnosperms (Figure 6). This finding indicates that the genome blocks containing these six *VQ* genes may have been preserved from ancestors of these gymnosperms. Additionally, through intragenomic collinearity analysis, we found that there are relatively more collinear blocks in *Ginkgo biloba*, including one *VQ* gene pair, which belongs to the segmental duplication events. Meanwhile, there are very few collinear blocks in the other two gymnosperms, and no *VQ* gene pairs were found (Figure S3 and Table 2). All these results suggest that most *VQ* genes and their duplication events in every gymnosperm mentioned in this study may have evolved independently in the latter stages. Furthermore, almost no collinear blocks and *VQ* gene pairs were observed between these three gymnosperms and *Arabidopsis* or rice.

### 2.5. Expression Patterns of VQ Genes in Different Tissues of Gymnosperms

**Figure 7:**
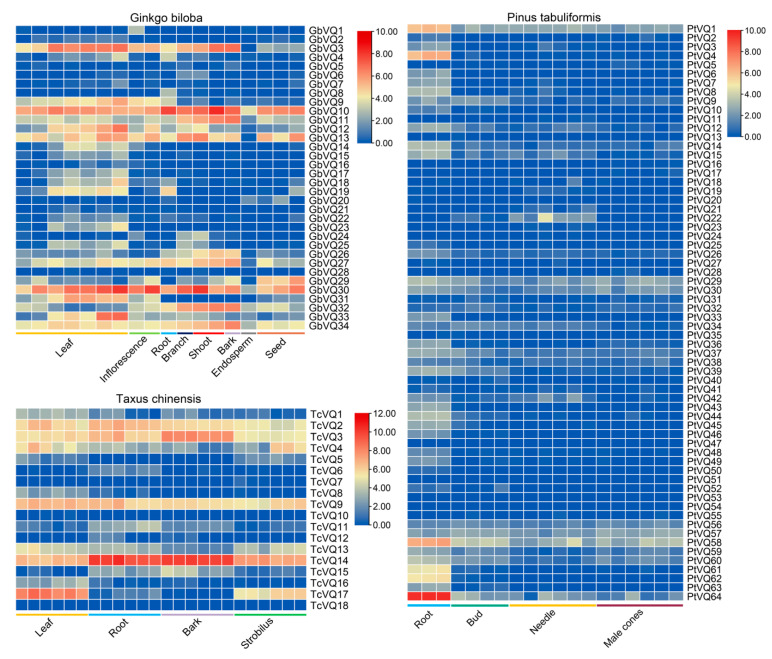
Expression level of *VQ* genes in different tissues of three gymnosperms. Expression levels of these *VQ* genes were obtained using RNA-seq data and measured using TPM values. Heatmap was generated using log2 (TPM + 1) values.

To characterize the expression patterns of *VQ* genes, the expression levels were analyzed in different tissues (eight for *Ginkgo biloba*, four for *Taxus chinensis*, and four for *Pinus tabulaeformis*) based on the published data in previous research to gain preliminary insight into their potential functions (Figure 7 and Table S5). Based on the TPM values, we found that 12 *VQ* genes (*GbVQ3*, *GbVQ10*, *GbVQ11*, *GbVQ12*, *GbVQ13*, *GbVQ26*, *GbVQ27*, *GbVQ29*, *GbVQ30*, *GbVQ32*, *GbVQ33*, and *GbVQ34*), 6 *VQ* genes (*TcVQ2*, *TcVQ3*, *TcVQ4*, *TcVQ9*, *TcVQ13*, and *TcVQ14*), and 15 *VQ* genes (*PtVQ1*, *PtVQ9, PtVQ14, PtVQ15, PtVQ29*, *PtVQ30*, *PtVQ34, PtVQ37*, *PtVQ38*, *PtVQ56*, *PtVQ57*, *PtVQ58*, *PtVQ59, PtVQ60*, and *PtVQ64*) were broadly and prominently expressed in different tissues in *Ginkgo biloba*, *Taxus chinensis* and *Pinus tabulaeformis*, respectively (Figure 7). In contrast, some *VQ* genes were only expressed in a few tissues, and some even had low or zero expression levels in different tissues (Figure 7 and Tables S6–S8). These *VQ* genes are denoted as playing different roles in regulating the plant growth and development of gymnosperms. These genes, which are expressed in multiple tissues, often play important roles in plant metabolism, disease resistance, and stress resistance [3,45,46], and they deserve further attention.

### 2.6. Phylogenetic Analysis of VQ Genes

**Figure 8:**
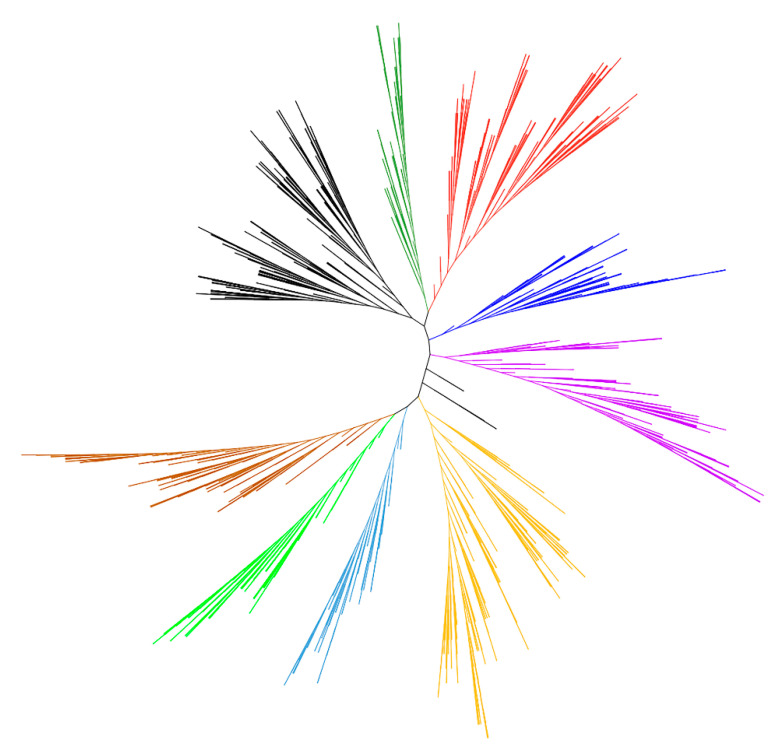
Unrooted phylogenetic tree of VQ proteins in 56 plant species. Multiple sequences alignment was performed using MAFFT (v7.511) software, and the phylogenetic tree was constructed via FastTree. Different colors indicate different groups, and these 2469 VQ proteins were clustered into nine subgroups.

To detect the evolutionary relationships and classification of the *VQ* family genes in 56 plant species, circular and unrooted phylogenetic trees were constructed with 2469 VQ proteins. In previous studies, according to structural characteristics, VQ proteins from different species have been clustered into 7 groups [6], 8 groups [3], 9 groups [47], and 10 groups [19]. In this study, we also built the phylogenetic tree for these 2469 VQ proteins from 56 plant species to explore their phylogenetic relationships, in which these proteins could be divided into nine groups (Figure 8 and Figure S4). *VQ* genes with unknown functions and pathways can often be inferred from species with close genetic relationships. Therefore, we also constructed a species evolution tree based on the taxonomy database of NCBI (Figure S5). The systemic clustering and biological classification of these 56 plant species can help us to better understand the functions and evolution of *VQ* genes in different plants. Moreover, *VQ* genes were clustered into 11 groups in three gymnosperms, which is basically consistent with the results of conservative motifs (Figure 5).

To further explore the evolutionary relationship of *VQ* gene between gymnosperms and angiosperms, as well as between monocotyledons and dicotyledons, nine species, including three gymnosperms (ginkgo, taxus, and Chinese pine) and six angiosperms (monocot: rice, wheat, and maize; dicotyledon: *Arabidopsis*, soybean, and tomato), were selected for further evolutionary analysis. The phylogenetic tree of VQ proteins from these species was drawn, and it showed that some branches are unique to gymnosperms, some are unique to angiosperms, and some have intersections, indicating that some VQ proteins may appear after the differentiation of angiosperms and gymnosperms (Figure S6). Similarly, monocotyledonous and dicotyledonous plants have their own independent and intersecting branches (Figure S6), which is consistent with previous research [3]. Interestingly, among widely expressed *VQ* genes, we found that four genes (*GbVQ3*, *GbVQ12, GbVQ33*, and *GbVQ34*) in *Ginkgo biloba*, one gene (*TcVQ2*) in *Taxus chinensis*, and three genes (*PtVQ29*, *PtVQ30*, and *PtVQ58*) in *Pinus tabulaeformis* were clustered with *AtVQ14, AtVQ9*, and *AtVQ5* (Figure S6), which were reported as influencing seed development, mediating salinity stress responses, and regulating plant defense, respectively [5,22,26]; three genes (*GbVQ26, GbVQ27,* and *GbVQ29*) found in *Ginkgo biloba*, three genes (*TcVQ13*, *TcVQ14*, and *TcVQ15*) in *Taxus chinensis*, and three genes (*PtVQ37*, *PtVQ38*, and *PtVQ39*) in *Pinus tabulaeformis* were clustered with *AtVQ22*, *AtVQ27*, and *AtVQ28* (Figure S6), which are related to plant defense and growth [18,48,49]. Among them, *GbVQ29*, *TcVQ13*, and *PtVQ38* are the only *VQ* genes in the collinear block shared by these gymnosperms, which indicates that these genes have a very conservative, important role and status in seed plants. Additionally, *GbVQ10*, *TcVQ3*, *PtVQ9*, *PtVQ59*, and *PtVQ60* were clustered with *AtVQ4*, which regulates disease resistance [19]; *GbVQ30*, *TcVQ9*, *PtVQ14*, *PtVQ15*, *PtVQ34*, and *PtVQ64* were clustered with *AtVQ15*, which negatively regulates osmotic stress tolerance [5,17] (Figure S6). These clusters can serve as references for the potential functions of these *VQ* genes to some degree.

The different characteristics of *VQ* genes in different types of plants can provide a molecular perspective for us to better understand plant evolution. All of these results provide important references for the study and utilization of *VQ* genes in plants, as well as for the evolutionary comparisons between gymnosperms and angiosperms.

## 3. Discussion

Plants are often affected by various environmental stresses throughout their lifetimes [50]. *VQ* family genes have been proven to play important roles in growth and development and responses to various abiotic and biotic stresses [2,5]. Therefore, it is of great significance to study the characteristics and functions of *VQ* genes in a wide range of plants. Up to now, *VQ* family genes have been identified and analyzed in multiple plant species. However, although there have been some reports on the structure and function of *VQ* genes, clear and intuitive feature information and knowledge of how to utilize these features are still limited in the plant kingdom, including bryophytes, gymnosperms, and angiosperms. Importantly, the *VQ* gene family of gymnosperms has not yet been reported. Thus, a comprehensive bioinformatics analysis of *VQ* genes in multiple plants, especially in gymnosperms, can provide an overall basis for evolutionary and functional studies of *VQ* genes.

### 3.1. Molecular Characteristics and Phylogeny of VQ Family Genes

In this study, we systematically identified 2469 *VQ* genes from 56 plant species. Gene numbers of VQ family are various among different plants. Compared to other plants, plants such as wheat and soybean contain abundant *VQ* genes (Table S2), which have undergone significant polyploidization and WGD events, respectively [3,51]. In *Ginkgo biloba*, *Taxus chinensis*, and *Pinus tabulaeformis*, 34, 18, and 64 *VQ* gene members were detected, respectively, but compared to other species, such as *Arabidopsis*, with 34 members [5], or rice, with 40 members [52], the *VQ* gene numbers in these three gymnosperms are far lower than expected considering their big genome size. Therefore, it can be concluded that the number of *VQ* genes has no necessary connection to the genome size, but it is related to species polyploidization and whole-genome replication events.

Except for the conserved VQ domain, VQ protein exhibits significant variability in other regions (Figure 1c). However, through MEME domain scanning, we also found some other motifs in different subgroups (Figure 5 and Figure S1), which may be related to the interaction, modification, and subcellular localization of these VQ proteins, affecting protein function. Although we have enriched multiple motifs in these VQ proteins, their specific functions still need further analysis. Many *VQ* family genes play important roles in growth and development, as well as biotic and abiotic stress, and interact with various proteins such as MAPK and WRKY. By conducting targeted editing and modification of these enriched candidate loci on the *VQ* gene, the functions and mechanisms of these motifs can be further revealed. Moreover, given the extensive biological functions of the *VQ* gene, this also has great potential in plant molecular precision breeding. Our study provides a method and idea for searching and utilizing conserved motifs and loci in gene families. The average length of VQ proteins is fewer than 300 amino acid residues (Figure 1d). This is one of the characteristics of the *VQ* family genes in plants, which helps us to identify them. Notably, some VQ proteins were found to be longer than 600 amino acids (Figure 1d), and these VQ proteins may contain other domains and participate in more diverse regulatory pathways.

Although the average GC content in the coding region of the *VQ* gene varies among different plants, the GC content in commelinids plants, including Poales and Musa plants, has significantly increased and is most prominent in Poaceae (GC content > 70%) (Figure 3). The *VQ* gene, as an ancient transcription regulatory cofactor in plants, plays an important role in the interaction between plants and environment. High GC content means better alkaline and high-temperature tolerance, allowing these *VQ* genes to remain relatively stable in environmental changes. Poaceae plants are the main food source for humans and many animals, and high GC content of their *VQ* genes is likely possessed by their ancestors and has been selected and fixed during evolution, which has become one of the characteristics of these plants. As for the deeper reasons why the GC content of *VQ* genes in Poaceae plants is prominent, further research is needed, and this is a very interesting and meaningful topic.

In the higher eukaryotes, intron-free genes are very common in their genomes [53]. In our study, based on the gene structure analysis, we found that most *VQ* genes in higher plants are intronless, no matter whether they are in angiosperms or gymnosperms, which is consistent with previous studies, including those of *Arabidopsis* [5], rice [6], tomato [28], apple [47], and wheat [3]. In contrast, lower plant moss is exactly the opposite [28] (Figure 2). Therefore, many studies speculated that the *VQ* genes in higher plants lost its intron during evolution [2,3,41]. However, we found that most *VQ* genes in tea variety ‘Longjing 43’ also contain introns and, at the same time, *Marchantia polymorpha*, known as a lower bryophyte plant, although there are only seven *VQ* genes in its genome, six of them (6/7) have no intron. So, the explanation of intron loss from the perspective of evolution history may need further investigation. Moreover, our results revealed that intron-containing *VQ* genes of gymnosperms are located in different subgroups, suggesting that these introns appear relatively independent, and this is also common in angiosperms [3,41]. Taken together, the identification of gene structure of *VQ* genes enriched our understanding of the evolution of introns in the plant kingdom.

Phylogenetic trees represent the genetic relationships between gene families from different species and reflect the similarity of protein-coding genes. To further understand the evolutionary relationships between these 2469 *VQ* genes from 56 plant species, we established a phylogenetic tree based on their protein sequences. They were classified into nine groups from our phylogenetic analysis (Figure 8 and Figure S4) and showed obvious evolutionary characteristics, such as the differentiation between angiosperms and gymnosperms and monocotyledons and dicotyledons. Additionally, in three gymnosperms, the *VQ* genes were classified into 11 groups, and in each group, they harbored similar type of motifs (Figure 5), suggesting a potential functional similarity. These results highlight the conservatism and diversity among *VQ* gene families of different plants. Based on the phylogenetic tree results, we can also use *VQ* genes with known functions to quickly search for homologous genes in specific plants. In theory, gene structure determines its function, and the more similar motifs shared between VQ proteins, the higher probability of their functional similarity. Therefore, systematic clustering combined with motifs analysis will help us to quickly identify homologous genes in different plants and make preliminary judgments on their functions. At the same time, further experiments are needed to verify their detailed functionality.

### 3.2. Expansion and Duplication Mechanism of VQ Gene Family in Gymnosperms

Genome replication events play an important role in expanding the size of the genome [54] and diversifying gene functions [55]. Chromosome fragment replication is considered to be the main expansion mechanism of gene family [56], and, thus, the evolutionary process can explain the number of specific *VQ* genes in a species, not the genome size. Previous research indicated that segmental duplication is the major mechanism contributing to the expansion of the *VQ* gene family in many angiosperms [3,4,41]. In the present study, we found that both tandem duplication and segmental duplication events of *VQ* genes exist simultaneously in *Ginkgo biloba*; only proximal duplication events exist in *Taxus chinensis*. Dispersed duplication, tandem duplication, and proximal duplication events exist in *Pinus tabulaeformis*; however, segmental duplication events only appear in *Ginkgo biloba* and account for a small proportion (Table 2). Segmental duplication is an important way of expanding the *VQ* gene family in angiosperms, while tandem duplication dominates the expansion of the *VQ* gene family in gymnosperms, which may be caused by different genetic and evolutionary mechanisms between angiosperms and gymnosperms. In addition, tandem duplication can reduce genetic instability caused by single-gene mutations or deletions and enhance plant resistance to environmental stresses, such as stress resistance and disease resistance. Given the importance of *VQ* genes, this is of great significance for ensuring the normal lives of gymnosperms.

The Ka, Ks, and Ka/Ks ratios of all *VQ* gene pairs were calculated to investigate whether *VQ* genes underwent selection pressure. The Ka/Ks values of most *VQ* gene pairs are less than 1.0, which demonstrates that purifying selection (Ka/Ks < 1) plays an important role in the evolution of *VQ* gene family in gymnosperms. Moreover, positive selection (Ka/Ks > 1) existed in some *VQ* genes (Table 2). Most *VQ* gene pairs are undergoing purification selection, while a few are undergoing positive selection, indicating that the *VQ* gene family in gymnosperms is in a stable dynamic evolution process.

Furthermore, only a few *VQ* gene pairs in collinear blocks were detected within and between *Ginkgo biloba, Taxus chinensis*, and *Pinus tabulaeformis.* For *Taxus chinensis* or *Pinus tabulaeformis*, there were even no *VQ* gene pairs detected in very few collinear blocks (Figure 6 and Figure S3). The results of synteny analysis indicate that the conservation degree of *VQ* genes among these three gymnosperms is low and their evolution process is relatively independent, which is similar to the difference between dicotyledons and monocotyledons in angiosperms [41], and this finding may be related to the fact that these three gymnosperms belong to different phytoclasses.

### 3.3. Expression Patterns of VQ Members in Gymnosperms

Previous studies have demonstrated that *VQ* genes are involved in regulating plant responses to biotic stresses, abiotic stresses, and growth and development [2,5]. The expression of many *VQ* genes showed various levels between different tissues and significant changes under pathogen, stress, or hormone treatments [3,5,6]. In this study, we detected the expression level of gymnosperm *VQ* genes in different tissues by analyzing RNA-seq data. The results showed that some *VQ* genes are not expressed in any tissues, some are only expressed in certain tissues, and some are widely expressed in different tissues. These widely expressed genes are usually associated with growth and development, hormone response, and plant defense [3,45,46], which requires further experimental verification in gymnosperms.

In *Arabidopsis*, *AtVQ14* is mainly associated with seed development, and its mutation produces small seeds [21,22]. Transgenic plants overexpressing *AtVQ5* displayed increased susceptibility to *B. cinerea* [5]. *AtVQ9* is strongly induced via NaCl treatment and negatively regulates the resistance to NaCl stress [26]. Compared to wild type plants, overexpression of *AtVQ17*, *AtVQ18*, or *AtVQ22* causes highly stunted growth of the transgenic plans [5]. AtVQ22/JAV1 can regulate JA-mediated plant defense and coordinate growth and defense [18]. Among these widely expressed *VQ* genes in gymnosperms, we found eight *VQ* genes (*GbVQ3, GbVQ12, GbVQ33, GbVQ34, TcVQ2, PtVQ29, PtVQ30*, and *PtVQ58*) as the candidate homologs of *AtVQ14*/*AtVQ9*/*AtVQ5,* nine *VQ* genes (*GbVQ26, GbVQ27, GbVQ29*, *TcVQ13*, *TcVQ14*, *TcVQ15*, *PtVQ37*, *PtVQ38*, and *PtVQ39*) as candidate homologs of *AtVQ22*/*AtVQ27*/*AtVQ28*, five *VQ* genes (*GbVQ10*, *TcVQ3*, *PtVQ9*, *PtVQ59*, and *PtVQ60*) as candidate homologs of *AtVQ4*, and six *VQ* genes (*GbVQ30*, *TcVQ9*, *PtVQ14*, *PtVQ15*, *PtVQ34*, and *PtVQ64*) as candidate homologs of *AtVQ15* (Figure 7 and Figure S6). This finding suggests that these candidate *VQ* genes may have similar functions and play an important role in growth, development, and response to external environmental stimuli. Among these genes, we found that TcVQ14 is almost identical to TcJAV3, which was reported to be a VQ motif-containing protein and homologous to AtVQ22/JAV1. TcJAV3-TcWRKY26 complex can regulate the expression of the downstream paclitaxel biosynthesis gene *DBAT* and participate in JA-mediated plant defense [57]. This finding indicates that our research results are trustworthy. In addition, gene duplication can produce gene function redundancy, and most of these repeated or collinear *VQ* genes showed almost identical expression patterns (Figure 7 and Table 2). Taken together, our study suggested that some *VQ* genes are involved in growth and development and participate in multiple life processes of gymnosperms.

## 4. Materials and Methods

### 4.1. Identification of VQ Genes in Multiple Plants

In the present study, genome sequence and annotation files of the 56 plant species were mainly collected from the Ensembl Plant database (http://plants.ensembl.org/index.html/, accessed on 2 March 2023) [58], NCBI plant genome database (ftp://ftp.ncbi.nlm.nih.gov/genomes/genbank/plant/, accessed on 2 March 2023), phytozome database (https://phytozome-next.jgi.doe.gov/, accessed on 2 March 2023) [59], and National Genomics Data Center (https://ngdc.cncb.ac.cn/, accessed on 2 March 2023). The Hidden Markov Model (HMM) profile of the VQ domain (PF05678) was obtained from the Pfam database (http://pfam.xfam.org/, accessed on 20 March 2023) [60]. The VQ family members were retrieved from plant protein sequences using the HMMSEARCH program of the HMMER (v3.0) software [61]. The online program SMART tool (http://smart.embl-heidelberg.de/, accessed on 25 March 2023) [62], the NCBI Conserved Domains Database (https://www.ncbi.nlm.nih.gov/Structure/cdd/wrpsb.cgi/, accessed on 25 March 2023) [63], and NCBI-BLAST (https://blast.ncbi.nlm.nih.gov/Blast.cgi/, accessed on 25 March 2023) were used to ultimately determine the *VQ* genes. Default parameters were adopted for all of the software mentioned above.

### 4.2. Molecular Features and Chromosomal Localization Analysis

The genome information, such as genome size, chromosome ploidy, and the number of total coding genes, were obtained from the Ensembl Plant database (http://plants.ensembl.org/index.html/, accessed on 2 March 2023) and other related published papers. GC content in the coding region, protein length, and sequence similarities of aligned sites of *VQ* genes were calculated via the Perl program (https://www.perl.org/, accessed on 2 March 2023). The relevant scripts and their input data can be downloaded from https://github.com/ywxkjtsd123/code (accessed on 15 September 2023). After the alignment of 2469 VQ protein sequences, the largest proportion of the same amino acids in each alignment site was calculated. The biophysical properties of the VQ proteins, including the peptide length, isoelectric point (pI), and molecular weight (MW), were estimated using the online program ExPasy (https://web.expasy.org/protparam/, accessed on 7 April 2023) [64]. The physical locations of the *VQ* genes on the chromosomes were visualized using the MapChart (v2.3) software [65]. The subcellular localization prediction tool DeepLoc-2.0 (https://services.healthtech.dtu.dk/services/DeepLoc-2.0/, accessed on 7 April 2023) [66] was used to predict the likely location of the *VQ* genes. The online tools NLStradamus (http://www.moseslab.csb.utoronto.ca/NLStradamus/, accessed on 15 September 2023) [67] and PSORT (https://www.genscript.com/psort.html/, accessed on 15 September 2023) [68] were used to predict the nuclear localization signals of proteins.

### 4.3. Conserved Motifs, Gene Structure, and Phylogenetic Tree Analysis

The conserved motifs of the VQ proteins were detected using the online program MEME (https://meme-suite.org/, accessed on 15 April 2023) with default parameters [69]. Conserved motifs were drawn using the TBtools (v1.123) software [70]. The exon and intron structures were determined via GFF file. After extracting their location information from the annotation file, the gene structures of the *VQ* genes were visualized using TBtools (v1.123) software. Based on the protein sequences, all multiple sequence alignments were carried out using the MAFFT (v7.511) software [71], using default parameters to study the evolutionary relationships and classification of the *VQ* genes. Depending on the alignment results, the phylogenetic tree was built using the FastTree 2 software [72] with default parameters. The online tool iTOL (https://itol.embl.de/, accessed on 15 April 2023) [73] was used to draw and adjust the phylogenetic tree. TBtools (v1.123) software was used to integrate phylogenetic trees, conserved motifs results, and gene structure results in three gymnosperms.

### 4.4. Gene Duplication and Collinearity Analysis

Gene duplication mainly includes segmental duplication, tandem duplication, dispersed duplication, and proximal duplication [42]. Segmental duplicates exist in collinear blocks. Tandem duplicates are defined as closely adjacent to each other on the same chromosome. Proximal duplicates are found on the same chromosome and are close to each other, but they are separated by several other genes. Dispersed duplicates occur on the same or different chromosomes, and they are neither close to each other nor within conserved collinearity blocks [42]. For the definition of tandem duplicates, we added the following concepts based on the literature: two or more *VQ* genes adjacent to each other within 200 kb can be defined as tandem duplication events [41]. To identify duplication events of the *VQ* gene family in the gymnosperms, the coding sequences (CDS) of all *VQ* genes were aligned using BLASTN with an E-value below 1 × 10^−15^. For the *VQ* gene with different isoforms, we selected the longest one for analysis. We used the following criteria to search for duplicate *VQ* gene pairs: both identity and coverage > 75% at the nucleotide level [3,74]. In addition, BLASTP (E-value < 1 × 10^−5^, top 5 matches) was used for sequence alignment, MCScanX [42] with the default parameters was used to identify collinear blocks within or between species, and the TBtools (v1.123) software and Circos program [75] were used to visualize the collinearity maps and exhibit segmentally duplicated *VQ* gene pairs. The values of nonsynonymous substitution rate (Ka) and synonymous substitution rate (Ks) of duplicated *VQ* gene pairs were calculated to evaluate the selection pressure using the KaKs_Calculator 2.0 [76] via the NG method. The relevant scripts and their input data can be obtained from https://github.com/ywxkjtsd123/code (accessed on 15 September 2023).

### 4.5. Gene Expression Patterns Analysis

The RNA-seq data of *VQ* genes in different tissues were obtained from previous research [36,37,77,78,79]. The sample name, accession number, and data source were listed in Table S5. Fastqc (https://www.bioinformatics.babraham.ac.uk/projects/fastqc/, accessed on 20 April 2023) was used for quality control, and Trimmomatic (v0.39) [80] was used for data filtering to obtain the clean reads. HISAT2 [81] was used for genome library construction and alignment. Transcript abundance was measured using TPM values. TPM values were calculated via StringTie2 [82] for *Ginkgo biloba* and *Taxus chinensis.* Due to the larger genome size and the limitations in computing resources, Bowtie2 [83] was used to build the index library and conduct sequence alignment, and RSEM (v1.3.3) [84] was used to obtain the TPM values for *Pinus tabuliformis*. A heatmap was generated using log2 (TPM + 1) values via TBtools (v1.123) software. Default parameters were used for all of the software used.

## 5. Conclusions

In this study, a systematic analysis was performed on the genome-wide identification, molecular characterization, phylogenetic relationship, and expression patterns of *VQ* genes in multiple plants, especially in gymnosperms. By analyzing the phylogenetic tree, conserved motifs, and duplication events, we have explained the functional divergences and expansion patterns of *VQ* genes. In conclusion, this study provides the first comprehensive and systematic analysis of the 2469 *VQ* genes identified in 56 plant species, and we also provided clear data that support the identification and evolution of the *VQ* gene in gymnosperms. The selection of candidate *VQ* genes could also provide a reference for future investigations in gymnosperms. Taken together, these findings can help us to better understand the evolution of *VQ* genes and provide a theoretical basis for further functional research and the practical utilization of *VQ* gene in various plants.

## Figures and Tables

**Table 1:** Summary of molecular characterization and subcellular localization of VQ proteins in three gymnosperms.

Species	Number	Motif Type						Length (aa)	MW (kDa)	pI	Subcellular Localization	
		LTG	FTG	VTG	MTG	YTG	FTA				Nucleus	Cytoplasm/Nucleus
*Ginkgo biloba*	34	23	8	2	-	1	-	283 ± 98	30.86 ± 10.25	7.67 ± 1.73	26	8
*Taxus chinensis*	18	13	3	1	-	-	1	165 ± 43	18.37 ± 4.81	8.55 ± 1.73	17	1
*Pinus tabuliformis*	64	36	22	2	4	-	-	260 ± 88	28.09 ± 9.10	8.50 ± 1.60	49	15

Protein length, MW; pI, mean ± SD.

**Table 2:** Duplication events of *VQ* genes in three gymnosperms.

Species	Gene 1	Gene 2	Duplication Type	Ka	Ks	Ka/Ks
*Ginkgo biloba*	GbVQ5	GbVQ6	Tandem	0.103073	0.129092	0.798447
	GbVQ5	GbVQ7	Proximal	0.095308	0.094741	1.005980
	GbVQ6	GbVQ7	Proximal	0.057674	0.059349	0.971771
	GbVQ14	GbVQ15	Tandem	0.044280	0.052344	0.845953
	GbVQ14	GbVQ16	Tandem	0.045420	0.033470	1.357020
	GbVQ15	GbVQ16	Tandem	0.062036	0.067561	0.918212
	GbVQ26	GbVQ27	Tandem	0.040319	0.041262	0.977136
	GbVQ2	GbVQ20	Segmental	NA	NA	NA
*Taxus chinensis*	TcVQ10	TcVQ12	Proximal	NA	NA	NA
	TcVQ11	TcVQ12	Proximal	0.098532	0.094428	1.043470
*Pinus tabuliformis*	PtVQ3	PtVQ4	Proximal	0.054239	0.179528	0.302121
	PtVQ5	PtVQ17	Dispersed	0.035409	0.034928	1.013750
	PtVQ6	PtVQ13	Dispersed	NA	NA	NA
	PtVQ6	PtVQ7	Proximal	0.004765	0.028118	0.169469
	PtVQ7	PtVQ13	Dispersed	0.142177	0.190426	0.746623
	PtVQ7	PtVQ8	Tandem	0	0	NA
	PtVQ8	PtVQ6	Proximal	0.004765	0.028118	0.169469
	PtVQ8	PtVQ13	Dispersed	0.179357	0.193909	0.924953
	PtVQ16	PtVQ5	Dispersed	0.009938	0.008609	1.154340
	PtVQ16	PtVQ17	Proximal	0.025090	0.044106	0.568853
	PtVQ18	PtVQ19	Proximal	0.032103	0.017421	1.842760
	PtVQ18	PtVQ20	Dispersed	0.035765	0.065227	0.548313
	PtVQ19	PtVQ20	Tandem	0.045874	0.053754	0.853421
	PtVQ21	PtVQ22	Tandem	0.098428	0.225648	0.436201
	PtVQ29	PtVQ30	Proximal	0.001346	0	NA
	PtVQ31	PtVQ32	Dispersed	0.001485	0.025303	0.058707
	PtVQ37	PtVQ38	Tandem	0	0	NA
	PtVQ44	PtVQ61	Dispersed	0.010209	0.005417	1.884470
	PtVQ44	PtVQ62	Dispersed	0.028367	0.030457	0.931388
	PtVQ44	PtVQ45	Tandem	0.025740	0.068154	0.377675
	PtVQ44	PtVQ46	Proximal	0.045012	0.063465	0.709246
	PtVQ45	PtVQ46	Tandem	0.059890	0.101127	0.592221
	PtVQ48	PtVQ49	Tandem	0	0	NA
	PtVQ48	PtVQ50	Proximal	0.004649	0.024470	0.189988
	PtVQ49	PtVQ50	Proximal	0.004947	0.026018	0.190129
	PtVQ54	PtVQ55	Dispersed	0	0	NA
	PtVQ56	PtVQ57	Tandem	0	0	NA
	PtVQ59	PtVQ60	Tandem	0	0	NA
	PtVQ61	PtVQ62	Tandem	0.031880	0.036130	0.882364
	PtVQ61	PtVQ45	Dispersed	0.026613	0.071133	0.374138
	PtVQ61	PtVQ46	Dispersed	0.048733	0.069510	0.701090
	PtVQ62	PtVQ45	Dispersed	0.021352	0.065554	0.325719
	PtVQ62	PtVQ46	Dispersed	0.058954	0.085332	0.690883

## Data Availability

The data presented in the study are available in the article and the Supplementary Materials. For further inquiries, you can directly contact the corresponding author.

## Supplementary Materials

The following supporting information can be downloaded at: https://www.mdpi.com/article/10.3390/ijms241914968/s1.
