# Supplementary material for: Large-Scale Identification and Characterization Analysis of VQ Family Genes in Plants, Especially Gymnosperms
Source: Int J Mol Sci. 2023 Oct 6;24(19):14968. doi: 10.3390/ijms241914968 (PMC10573558; doi:10.3390/ijms241914968)
Supplement: Supplementary file 1 [file ijms-24-14968-s001.zip › ijms-2584089-Supplementary Figures S1-S6.pdf]

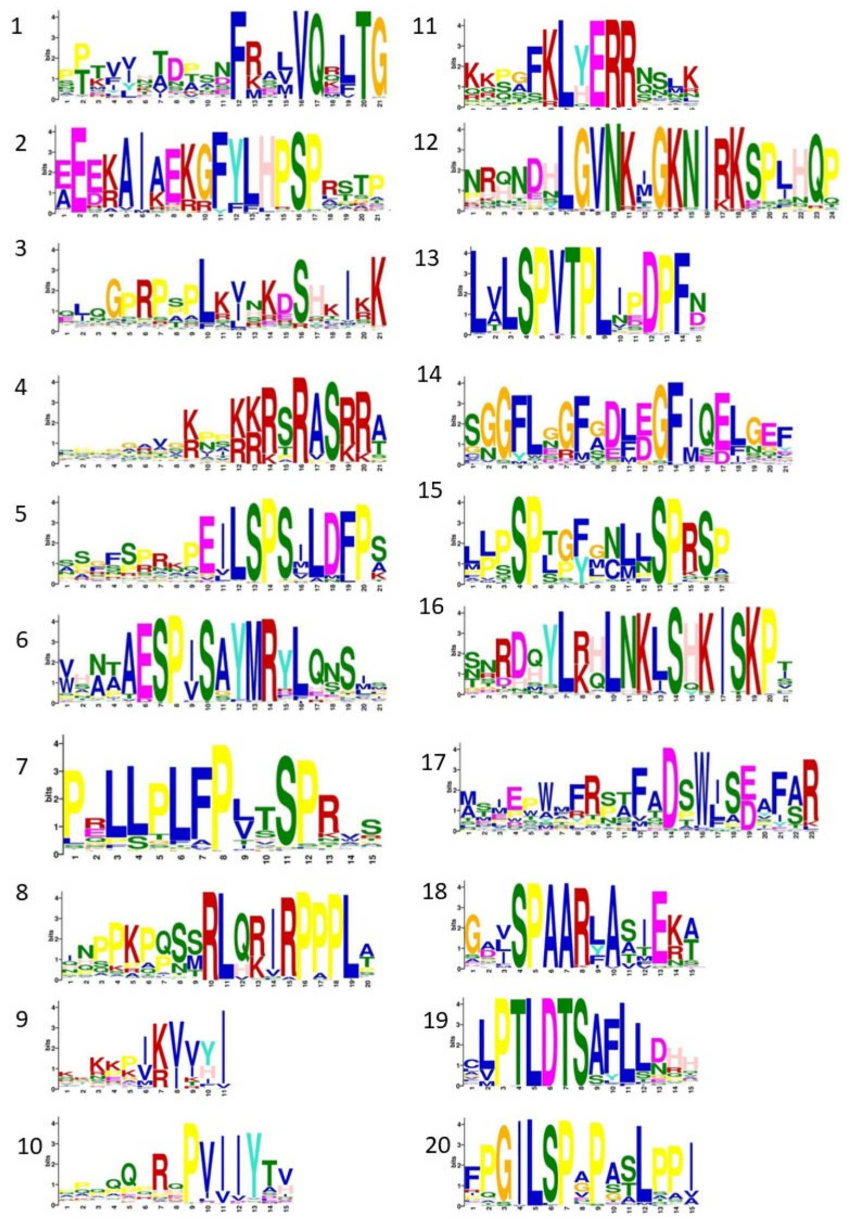

Figure S1. Top 20 conserved motifs of VQ protein in 56 plant species. The MEME online program (https://meme-suite.org) (Bailey et al., 2015 [1]) was used to identify motifs in the 2469 VQ protein sequences.

>AtVQ22/JAV1
MANPNEWSQFYNNNQTFFTTSTTASTAVTTTTAGDTTSIDSRLSPETGRVTKPTRRRSRASRRTPTTLLNTD
TSN<u>FRAMVQQYTG</u>GPSAMAFGSGNTTSAFSLTSSSDPSAGSSQQAPWQYNFQPHAPLQPPQRPYMFSLNN
VNPVVGYSNMNNPNTMVSGVFGTVDGSGGGGSAPSSKEATNSNSSSSRLQ

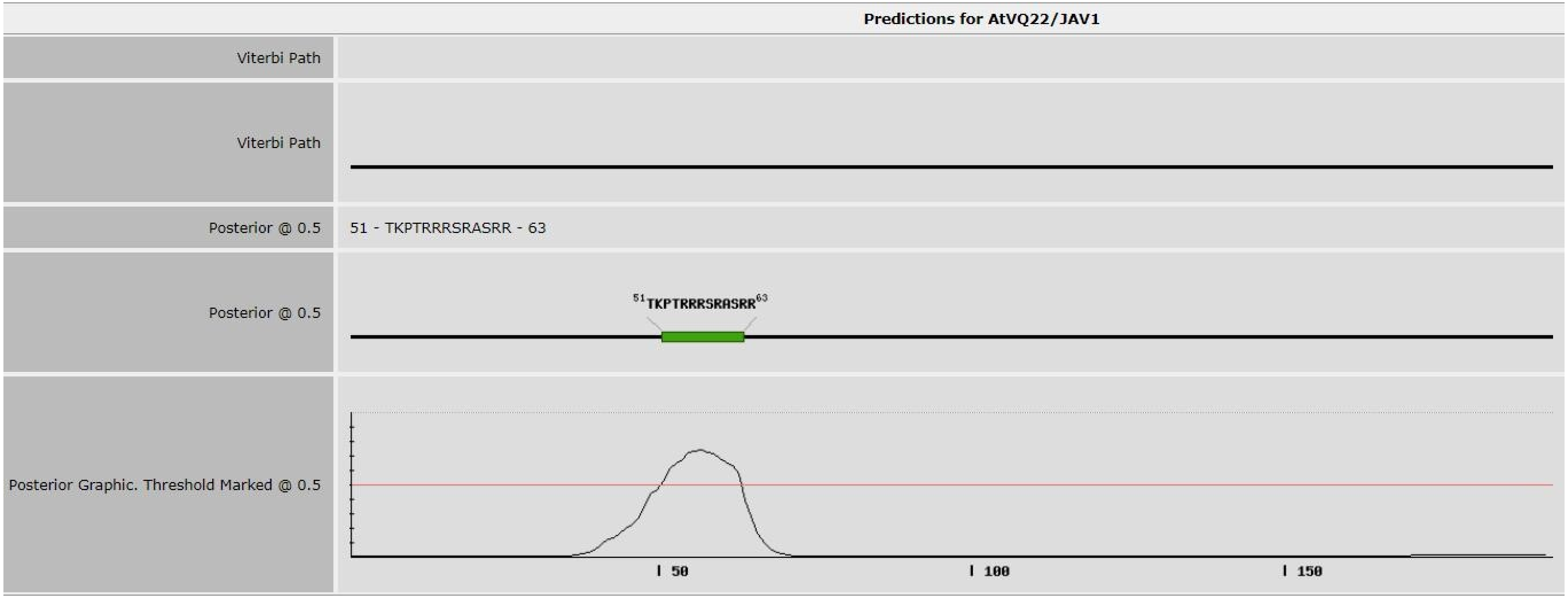

[NUCDISC: discrimination of nuclear localization signals]

```
pat4: none
pat7: PTRRRSR (5) at   53
bipartite: none
content of basic residues:   6.8%
NLS Score: -0.04
```

>OsVQ25
MAAMSDTGSSLAQWAELYHDASAAHGGVVANGAAAAATSPASPAGSTGGSPTRAPGVEGPRVGKPARRR
SRASRRAPVTLLNTDTTNFRAMVQQFTGIPAPPAGAFAGPGGVPVINFGSDYGFTGAVLPFSDHLQPRRPTF
QDHQQLLRPQQQYTGAPFGYGNLQQAGGAGTGAGDMFSHALSSAEDRLLLQSLQSAQMPTSAANHSAN
GYFA

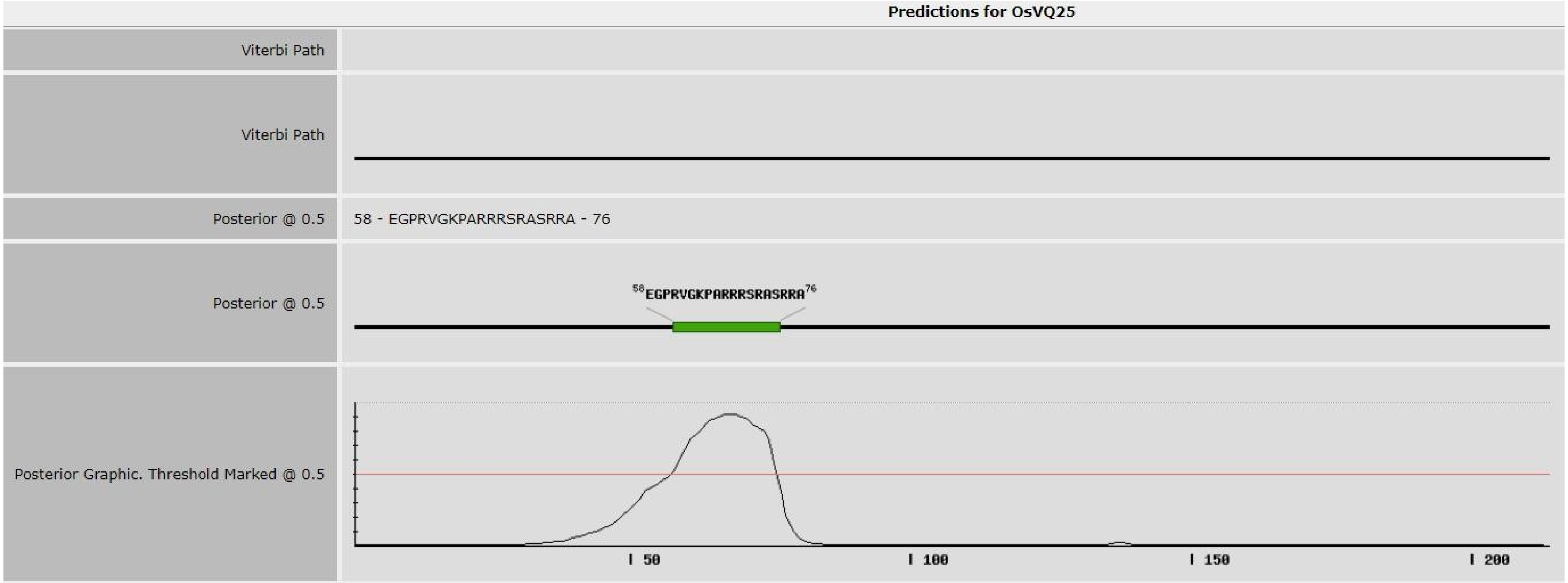

NUCDISC: discrimination of nuclear localization signals

```
pat4: none
pat7: PARRRSR (5) at   65
bipartite: none
content of basic residues:   6.6%
NLS Score: -0.04
```

Figure S2. Nuclear localization signal prediction.

The online tools NLStradamus (http://www.moseslab.csb.utoronto.ca/NLStradamus) and PSORT (https://www.genscript.com/psort.html) were used to predict the nuclear localization signals of VQ protein. The yellow area is the nuclear localization signal region, and the underlined area is the VQ motif.

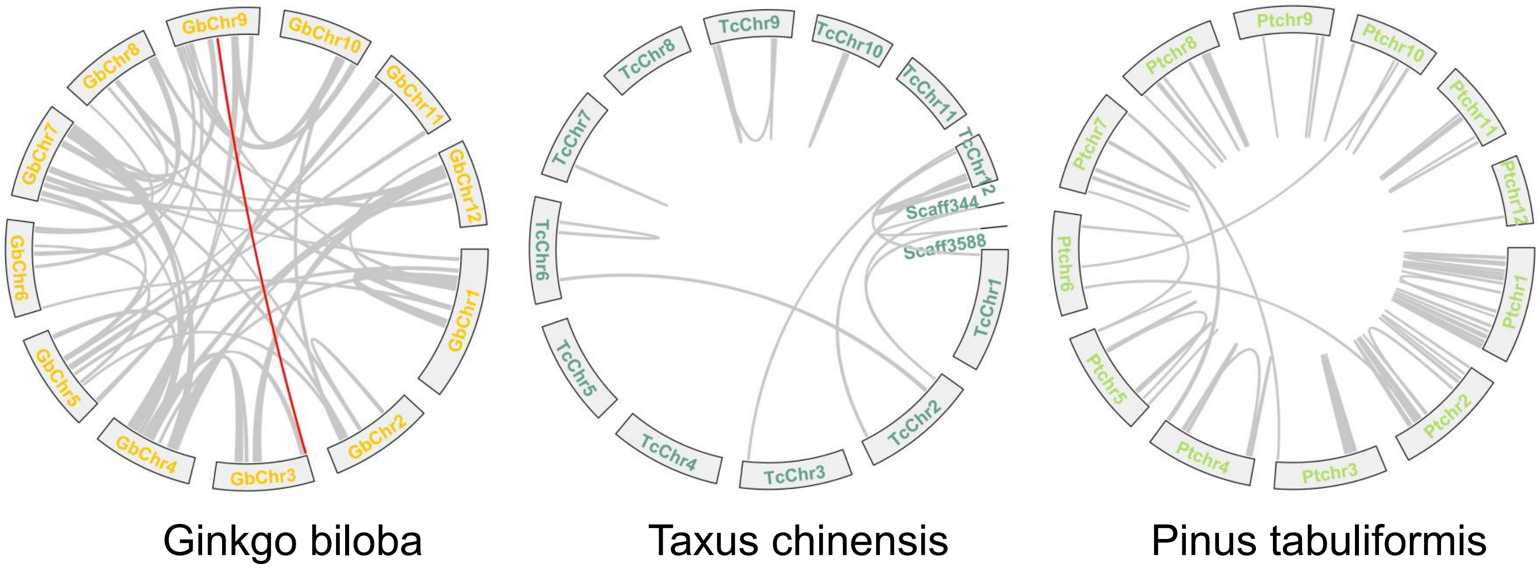

Figure S3. Collinearity analysis in gymnosperms.

MCScanX (Wang et al., 2012 [2]) was used to identify collinear blocks in gymnosperm genomes. The red lines highlight the collinear *VQ* gene pairs.

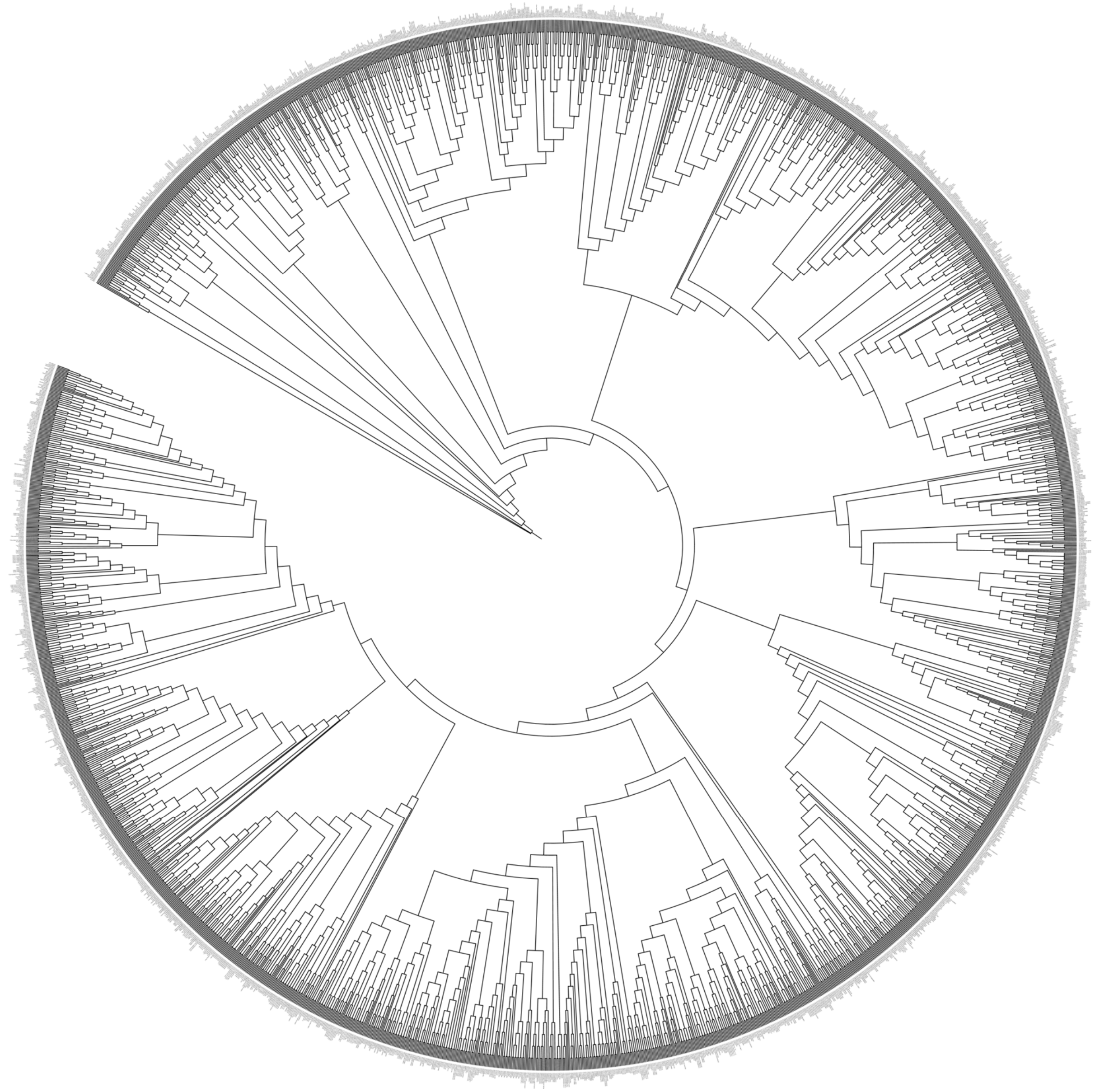

Figure S4. Circular phylogenetic tree of VQ proteins in 56 plant species.

Multiple sequence alignment was performed using MAFFT (v7.511) software (Katoh and Standley, 2013 [3]). The phylogenetic tree was calculated via FastTree 2 (Price et al., 2010 [4]) and drawn via iTOL (https://itol.embl.de) (Letunic and Bork, 2021 [5]).

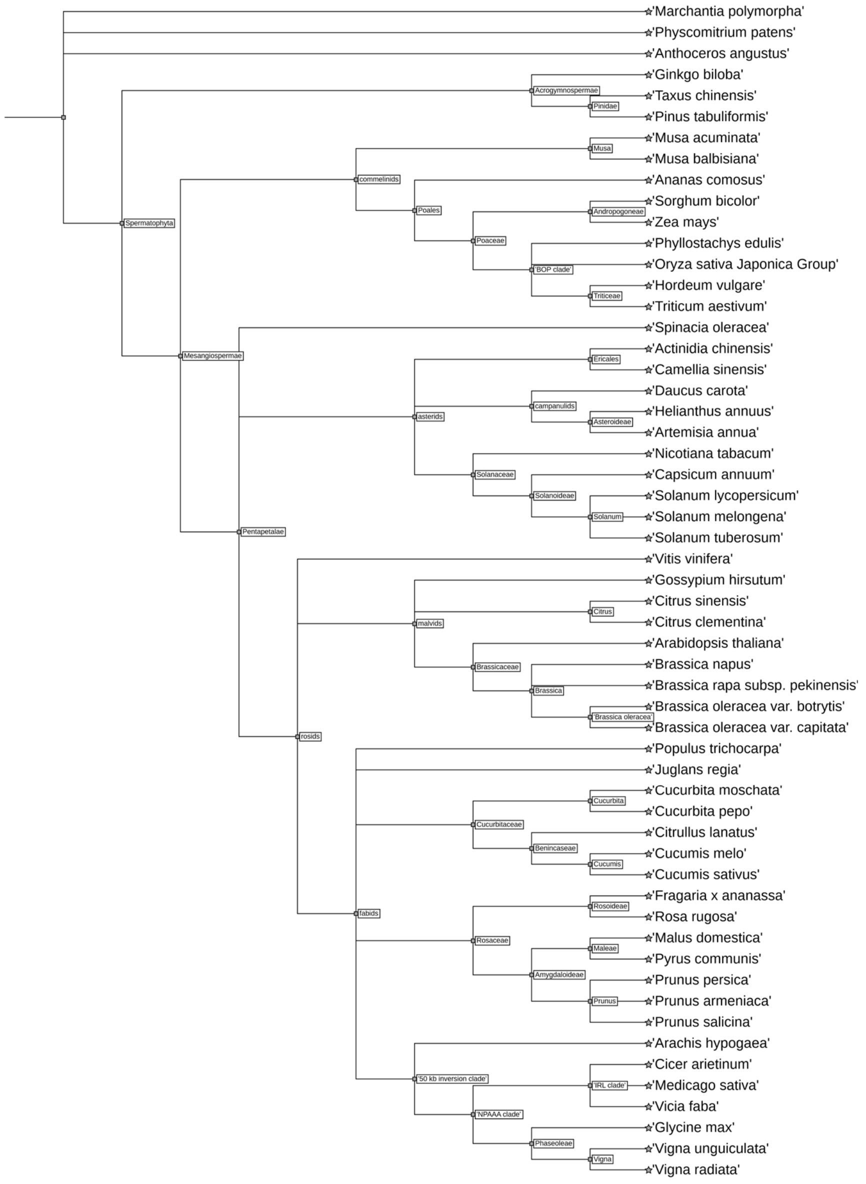

Figure S5. Taxonomy classification for 56 plant species. .

Taxonomy classification was performed in the NCBI Taxonomy Database (https://www.ncbi.nlm.nih.gov/taxonomy).

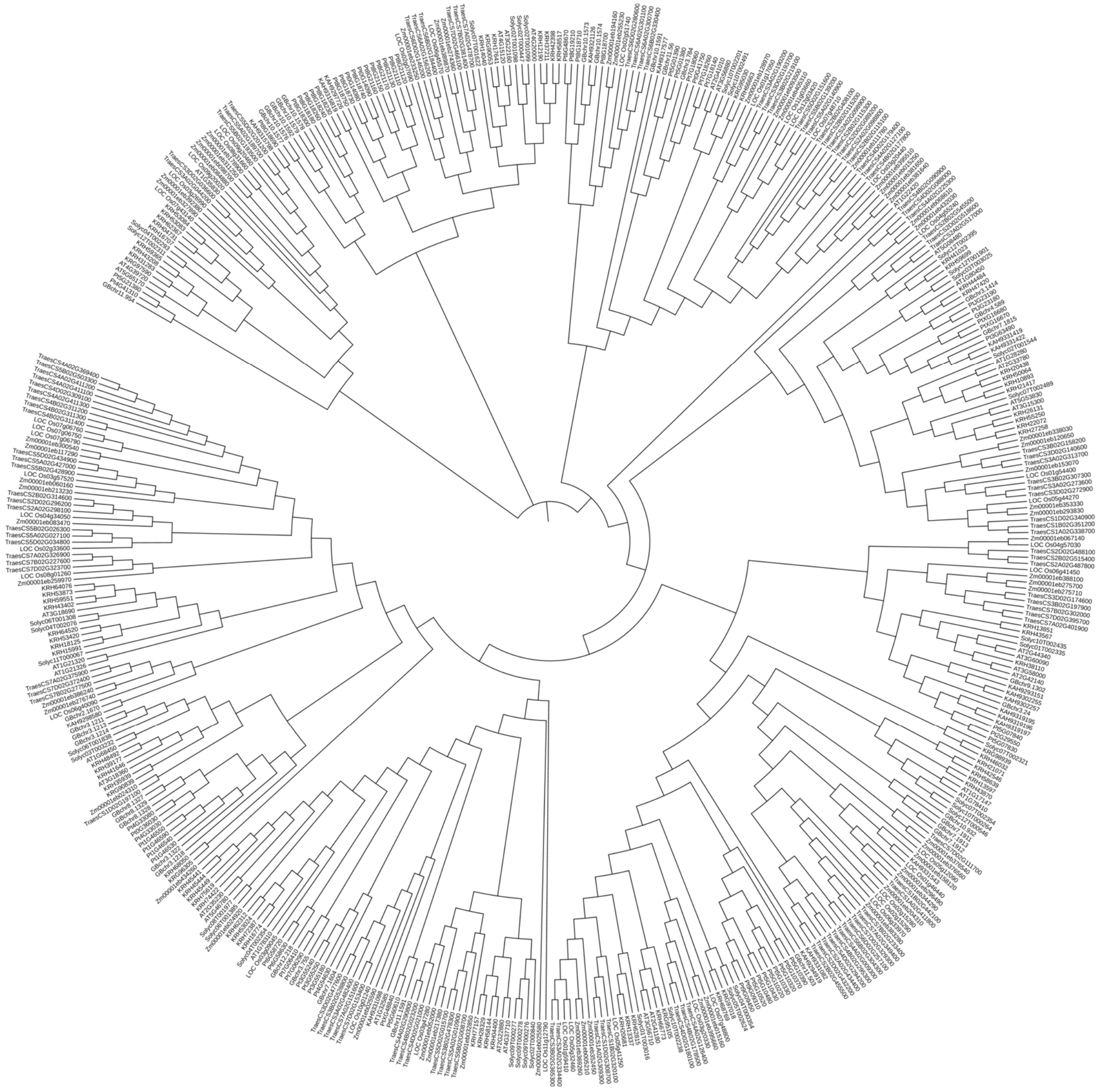

Figure S6. Circular phylogenetic tree of VQ proteins in three gymnosperms and six angiosperms.

Multiple sequence alignment was performed using MAFFT (v7.511) software (Katoh and Standley, 2013 [3]). The phylogenetic tree was calculated via FastTree 2 (Price et al., 2010 [4]) and drawn via iTOL (https://itol.embl.de) (Letunic and Bork, 2021 [5]).

References

1. Bailey, T.L.; Johnson, J.; Grant, C.E.; Noble, W.S. The MEME Suite. *Nucleic Acids Res.* **2015**, *43*, W39–W49. https://doi.org/10.1093/nar/gkv416.
2. Wang, Y.; Tang, H.; Debarry, J.D.; Tan, X.; Li, J.; Wang, X.; Lee, T.H.; Jin, H.; Marler, B.; Guo, H.; et al. MCScanX: A toolkit for detection and evolutionary analysis of gene synteny and collinearity. *Nucleic Acids Res.* **2012**, *40*, e49. https://doi.org/10.1093/nar/gkr1293.
3. Katoh, K.; Standley, D.M. MAFFT multiple sequence alignment software version 7: Improvements in performance and usability. *Mol. Biol. Evol*. **2013**, *30*, 772–780. https://doi.org/10.1093/molbev/mst010.
4. Price, M.N.; Dehal, P.S.; Arkin, A.P. FastTree 2—Approximately maximum-likelihood trees for large alignments. *PLoS ONE* **2010**, *5*, e9490. https://doi.org/10.1371/journal.pone.0009490.
5. Letunic, I.; Bork, P. Interactive Tree Of Life (iTOL) v5: An online tool for phylogenetic tree display and annotation. *Nucleic Acids Res*. **2021**, *49*, W293–W296. https://doi.org/10.1093/nar/gkab301.
